# A Novel Acceleration Signal Processing Procedure for Cycling Safety Assessment

**DOI:** 10.3390/s21124183

**Published:** 2021-06-18

**Authors:** Emanuele Murgano, Riccardo Caponetto, Giuseppina Pappalardo, Salvatore Damiano Cafiso, Alessandro Severino

**Affiliations:** 1 Dipartimento di Ingegneria Elettrica Elettronica e Informatica, University of Catania, 95125 Catania, Italy; riccardo.caponetto@unict.it; 2 Dipartimento di Ingegneria Civile e Architettura, University of Catania, 95125 Catania, Italy; giuseppina.pappalardo1@unict.it (G.P.); dcafiso@unict.it (S.D.C.)

**Keywords:** wavelet decomposition, Dynamic Time Warping, bicycle, GPS tracking, traffic conflict

## Abstract

With the growing rate of urban population and transport congestion, it is important for a city to have bike riding as an attractive travel choice but one of its biggest barriers for people is the perceived lack of safety. To improve the safety of urban cycling, identification of high-risk location and routes are major obstacles for safety countermeasures. Risk assessment is performed by crash data analysis, but the lack of data makes that approach less effective when applied to cyclist safety. Furthermore, the availability of data collected with the modern technologies opens the way to different approaches. This research aim is to analyse data needs and capability to identify critical cycling safety events for urban context where bicyclist behaviour can be recorded with different equipment and bicycle used as a probe vehicle to collect data. More specifically, three different sampling frequencies have been investigated to define the minimum one able to detect and recognize hard breaking. In details, a novel signal processing procedure has been proposed to correctly deal with speed and acceleration signals. Besides common signal filtering approaches, wavelet transformation and Dynamic Time Warping (DTW) techniques have been applied to remove more efficiently the instrument noise and align the signals with respect to the reference. The Euclidean distance of the DTW has been introduced as index to get the best filter parameters configuration. Obtained results, both during the calibration and the investigated real scenario, confirm that at least a GPS signal with a sampling frequency of 1Hz is needed to track the rider’s behaviour to detect events. In conclusion, with a very cheap hardware setup is possible to monitor riders’ speed and acceleration.

## 1. Introduction

Cyclists represent 8% of all road deaths in the European Union with about 2000 fatalities in 2016 [1]. While small in proportion with motorized vehicles, they are generally unprotected and vulnerable in traffic and, therefore, encountered in severe injuries or fatal consequences. Studies report that “the risk of a serious injury per kilometre travelled is 23 times higher for cyclist than for car drivers” [2]. Big disparities in terms of accident exist between countries depending to the use of cycling as mode of transport, i.e., the Netherlands (19%), the Denmark (15%) and the Germany (12%) [3].

In literature, some naturalistic cycling studies investigated various aspect of mobility and cycling behaviour, such as UDRIVE [4] to identify the behavioural patterns in cyclists’ interactions, InDev (In-Depth understanding of accident causation for Vulnerable road users) Project [5] where a review of current studies about Safety Critical Events (SCEs) involving cyclists was analysed, or the Australian project BIKEALYZE [6] where a naturalistic cycling study was performed. More specifically, the identification of safety-critical events was performed via self-reporting, manual review of video footage and indicators collected via the naturalistic data. On the contrary, in other studies, participants had an active role to indicate any SCE they experienced via a push-button on the vehicle [7] or in a smartphone app [8].

However, none of them has capability to self-detect accidents. Up to now, only few studies have been conducted to take over traffic conflicts based on motion patterns. Generally, they used speed [9], acceleration [9,10] and rotation patterns [9] to detect bicycle accidents. The application of a smartphone is very widespread and the motion data collected by the phone sensors such as velocity from GPS and acceleration from accelerometers. They have been used to identify an abnormal motion that occurs in the event of an accident [10,11].

Often, non-motorized vehicle based accidents are often not reported to the police, and hence do not appear in official statistics. Only 50% of traffic accidents in which cyclists are hospitalized are reported in European police data. This underreporting can lead to a severe bias in accident statistics, as the differences between official reported bicycle crashes and cycling injury data which is collected by hospital’s record. In addition, the under-reporting of the number of crashes (the numerator) recorded with respect to crash rates, the under-reporting also suffers from a less reliable exposure factor (denominator, e.g., number of cyclists, trips, or kilometres of cycling in the relevant group) compared to the estimation rate for the motorized traffic. Consequently, the estimated rates of injuries, and crashes, are both under-estimates of the magnitude of the problem, and less reliable than the estimates for drivers and car occupants [12]. Observational studies usually do not suffer from such bias and the traffic conflict technique (TCT) which is a promising methodology of field observations to quantitatively describe the interactions between road users those are involved in a critical event for safety, not only in the occurrence of a crash [13]. With this technique, it is possible to record safety critical events (SCEs) as “situations that require a sudden, evasive manoeuvre to avoid a crash or to correct for unsafe acts performed by the driver himself/herself or by other road users” [14]. Behaviour in traffic is recorded in its entirety, so there is no systematic underreporting for several types of incidents or accidents.

### Problem Statement and Methodological Approach

The goal of our study is to analyse data needs and capability to identify SCEs in cycling during riding tests in urban environment. For this reason, the dynamic of an instrumented bicycle was monitored with cameras, accelerometers and two GPS systems working at different acquisition frequencies, as well. Three distinct research questions are treated in the paper:

- What equipment can provide suitable measures and data?
- How to account for the noise induced in the sensor’s signals?
- What ride analytics are informative to identify critical events?

Noise affects the acquisitions and can hide some hard breaking, Therefore, raw data must be treated by applying tailored denoising and filtering procedures. The present work proposes a filtering approach to reduce noise from speed data collected by GPS devices to leverage the data collection capabilities of smartphones and generate information about the bicyclist behaviour. The processing procedure was firstly applied in a controlled calibration test in order to evaluate its overall efficiency or on the basis of its parameters. This first acquisition was carried out in a closed track by two cyclist riding at different speeds with controlled braking and accelerating phases. Raw data was analysed and compared to the reference values to identify the more suitable acquisition system and filtering processes. Results and the proposed methodology (calibrated in the test track) is validated in a real traffic scenario. The paper is organized as follows. The section “GPS Data and Cycling” presents the state of art relating to the use of GPS in the context of cycling. The design of the field experiment carried out for the signal processing algorithms to report the results are presented in section “Material and Method”. Finally, the results are depicted in two different scenarios: a calibration test performed to tune the processing procedure and a real scenario to consolidate the process. In the last section, the paper concludes with a summary and discussion and identifies limitations and areas of further work.

## 2. GPS Data and Cycling

Since 1990s, GPS data on location and speed have been collected for transport analysis and for evaluating system performance such as measuring traffic flow levels, analysing travel behaviour and estimating route choice models [15,16]. In May 2016, Google announced the availability of GNSS (Global Navigation Satellite System) raw measurements from Android 7 and now the standard formats, such as RINEX or NMEA, are available on the Android platform, as well. In smartphone, the GNSS raw data processing benefits from augmentation data (e.g., EGNOS) and tight integration with cellular, WIFI and Bluetooth (data fusion) that improve the accuracy and availability of the final position. Moreover, the NMEA string includes other useful information apart of PVT (Position, Speed, Time) like the heading [17]. By exploiting all its available data, several study have focused on the accuracy of estimating driving parameters (e.g., speeding, harsh braking/acceleration, harsh cornering) from smartphones probes [18,19]. Unfortunately, these studies are related only to motor vehicle applications and we can find few studies for bicycle [7,10,20]. In [7] GPS data are used only for the localization of the event, in [20,21] smartphone GPS coordinates are used to compute instantaneous speed at time *i*.

Although the GPS speed is enough accurate, it requires to be processed to define the riding characteristics (e.g., acceleration) and the accelerations deduced therefrom are affected by noise and depend on the satellites visibility and acquisition frequency [22]. Furthermore, hardware constraints have to be taken into account: typical GPS locations (i.e., E, N coordinates) are sampled limiting the acquisition in every few seconds, nevertheless generating hundreds of data points per individual journey, generating an extremely wide dataset for a complete study. Since a continuously operating GNSS chipset will drain the battery, the duty cycle of 4 or more seconds is applied to improve power consumption.

Generally, in cycling GPS data was only used to determine the model of cyclists’ route [21,23]. Moreover, the chances to collect GPS data increased significantly with the growth in sports and fitness apps, used by a huge number of users. Apps like Endomondo, MapMyRide, Strava, Garmin Connect, TrainingPeaks, Komoot, RideWithGPS, are widely used by cyclist for tracking sport activities. For detailed studies, this data present two types of limitations: the first depends on the aggregated information, the second on the sampling frequency. Because these apps have only aggregated socio—demographic informations, there is limited scope to analyse the importance of basic factors like age and gender in route planning, journey length or purpose. Being aimed exclusively at route profiles, GPS data are sampled from three up to five seconds in order to minimize their dimension for the storage requirements. In addition, the point data from Bike Share Programmes (BSP) stations are becoming input data for the study of cyclist safety [24,25,26], and the correlation with weather conditions [27] and land use [28,29].

## 3. Materials and Methods

**Figure 1:**
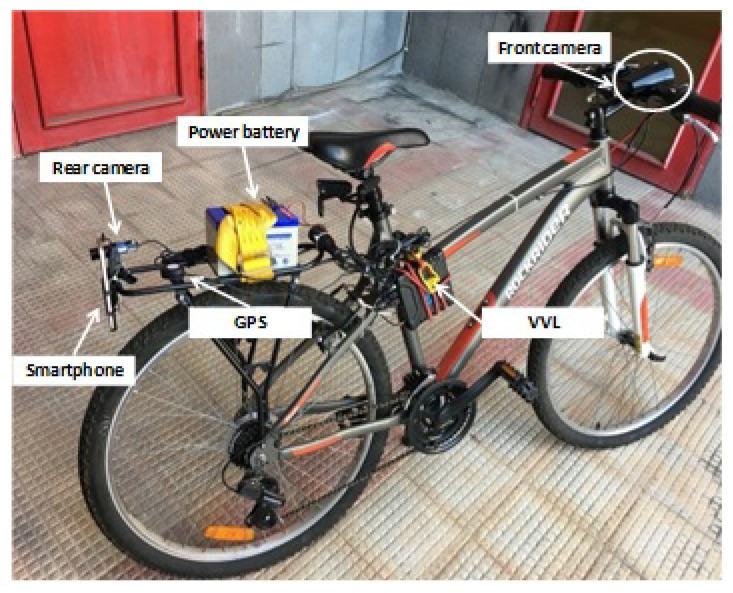
Experimental setup.

When GNSS receiver is used to analyse in detail biker riding analytics in detail, other than the accuracy acquisition frequency is also an issue because of the lower cycling speed 3m/s–7m/s and event duration, 2s–4s, compared to motorized vehicles. Therefore, an instrumented bike equipped with a different GPS, encoder and video systems to carry out experiments in controlled conditions, Figure 1.

**Figure 2:**
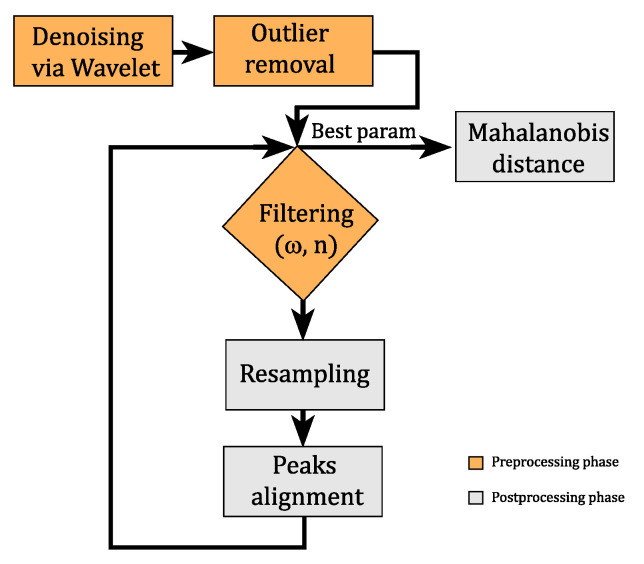
Flowchart of the signal processing procedure.

Two different GPS systems have been used. The Video VBOX Lite (VVL) is a standard GPS single frequency with an accuracy of ±3 m in the position and ±0.1 km/h in the speed. The Samsung S7 smartphone GPS apply an AGPS correction providing better accuracy in the positioning, expecially with limited satellites view (e.g., urban canyon) but with limited improvements in speed data. The VVL provided GPS data, velocity and acceleration, synchronized with a video recording, with a sampling frequency of 10 Hz. This system will be referred in the following as VBOX. One camera recorded the front view of the cyclist while the other one was at forward facing. The videos were recorded with a resolution of 720 × 576 pixels at 25 frames per second [30]. The Samsung S7 smartphone collected GPS data with a frequency of 1 Hz, and referred in the following as GPS. The GPS NMEA string was provided by the android app “HiperIMU” and referred in the following as “HIMU”. With the same app, it was possible to acquire acceleration from the smartphone accelerometer which has a variable sampling frequency in the range f_s,HIMU_ = 40 Hz–45 Hz. Tacheometer was also installed to record directly the velocity from the wheel rotation. The speed acquired from tacheometer was sampled at frequency of 2 Hz and it has been used as reference data for all the successive analysis. Therefore, the acquired speed from the odometer, the VBOX and the GPS, are available for studying the cyclists’ behaviour exploiting the analysis procedure depicted in Figure 2. The aforementioned flowchart shows the steps to choose the best cut off frequency ω and filter order *n*. All the routines related to the flow chart blocks have been implemented in MATLAB.

### 3.1. Signal Denoising

From raw signals, the background noise has been removed applying the wavelet transform [31]. The wavelet approach was chosen because it permits to analyses and process the signals both in time and frequency domain. Usually, for signals analysis the Fast Fourier Transform (FFT) is widely used. The FFTs allows to identify all the frequencies characterizing the considered signal that it allows to define the cut-off frequency to filter it. A drawback of the FFTs is related to its capability to detect when a specific frequency appears in time. This drawback can be partially solved applying the Short-Time Fourier Transform (STFT) that evaluates the Fourier transform on finite time interval: in this way it is possible to detect when a frequency appears in time [32]. In general, a non-stationary signal is characterized by a long period behaviour at low frequencies, called “trends”, and short period behaviours at high frequencies, called “anomalies”. In the following analysis a 5-level wavelet decomposition has been applied to obtain the wavelet coefficients. Furthermore, a hard thresholding has been performed using the universal threshold rule, i.e., $\sqrt{2\ln\left(length(x)\right)}$ to obtain the decomposition structure, where length(·) evaluates the number of samples for its argument.

### 3.2. Outliers Removal

Outliers are those samples which seem to be inconsistent with the main trend of a signal. They might be peaks, discontinuities, saturation, sensors failures and so on. To analyse correctly a signal, they should be removed in a proper way (i.e., without changing the remaining part of the signal) [33]. Generally, they could appear in groups or randomly isolated and, in the latter scenario, an interpolation could be applied. On the contrary, if they appear in group, it is better to divide the datasets in several intervals because the interpolation procedure is meaningless.

When isolated outliers occur, the most used technique to remove them is the so called ‘3−σ edit rule’ [34]. Supposing to have a normal distribution, for each sample $x_{i}$, the normalized distance $d_{i}$ from the estimated mean is evaluated as:

(1) $$d_{i}=\frac{x_{i}-\bar{x}}{\sigma_{x}}$$

where $\bar{x}$ is the mean of the signal and $\sigma_{x}$ is its standard deviation. If $\left|d_{i}\right|>3$, i.e., $\left|d_{i}\right|>3\sigma_{x}$, it means that this sample belongs to the tail of the signal distribution; hence, this sample is statistically irrelevant and it can be removed interpolating the previous and the next samples. The probability that the aforementioned condition occurs is about 0.27%.

In particular, in the bicycle signals analysis, more attention needs to be paid because the outliers could be the hard breaking that occur and strongly change the signal trend. For this reason, before and after performing the outlier removal technique, data have been deeply investigated in order to verify if an outlier swamping occurs, i.e., a valid sample is classified as an outlier. It has been found from visual inspection that the 3−σ edit rule does not corrupt the signal trends, without losing information.

### 3.3. Signal Filtering

After the background noise and outlier removals, the next step is the signal filtering. Two degrees of freedom are specified, i.e., two parameters (cut-off frequency $\omega_{c}$ and order *n*) that can be changed to filter differently the signals and evaluates how one parameter changes the correlation coefficient. Vehicle manoeuvres are typically characterized by using much lower frequency, since their durations tend to be in the order of seconds [35]. Low-pass Butterworth filter [36] has been chosen for this operation and its frequency response is defined as:

(2) $$G\left(\omega \right)=\frac{1}{\sqrt{1+{\left(\frac{\omega }{\omega_{c}}\right)}^{2n}}}.$$

where $\omega_{c}$ is the cut-off frequency, *n* is the filter order and $\omega_{n}=\omega /\omega_{c}$ is the normalized frequency.

Analysing the frequency response of (2) it is possible to notice that increasing the order of the filter implies a decrease in the signal band: ideally, if n→∞ all the frequencies greater than $\omega_{c}$ are removed. This choice is unrealizable both in real analogue implementation and is also computationally expensive. For the cycling case study an order greater than 10 is not necessary. Besides, the filter is built with a zero-phase digital filtering [37] that processes the signal in both forward and backward directions to avoid delays in the filtered signal. The filtering procedure is performed changing both cut-off frequency and filter order. For the latter, it changes from 1 up to 10 with a step-size of 1, while the former starts from 0.04 Hz up to half of the sampling signal frequency with a step-size of 0.05 Hz: in this way it is possible analysing those frequencies where significant information is present.

### 3.4. Resampling Procedure and Peaks Alignment

The applied filter procedure requires the design of a least-squares linear-phase FIR, which minimizes the weight that integrated with squared error between an ideal piecewise linear function and the response of the designed filter for a fixed bandwidth [38]; then, a rate change, i.e., upsampling, is applied. The interpolated signal is then convoluted with a FIR impulse response and finally the result of this operation is downsampled [39]. All these steps can apply via MATLAB command resample [40], which also allows to fix a non-integer-order factor to downsample a signal. This procedure has been performed after the filtering phase in order to avoid noise or other artefacts. Once that all signals have been resampled at the same length, a peak alignment procedure has been performed. In literature, for speech signals and more recently for vehicle signals [41,42], a procedure able to stretch two signals in order to align their peaks is the Dynamic Time Warping (DTW) [43]. Given two signals, *x* and *y*, which have similar features but placed in different time locations (e.g., peaks in a signal which occur at different time instants, due to sensor delay or different sampling frequency), the DTW distorts them fixing a new temporal scale in which they show the features in the same time instant. In particular, this algorithm tries to minimize the Euclidean distance $d_{\left(x_{i};y_{i}\right)}$ between each sample of the two signals: to realize this new temporal scale, each sample can be repeated several times to build the new reference axis, e.g., x(1) has to be repeated 5 times, y(5) 10 times and so on. It must be noticed that this new temporal scale allows to align the signals but cannot be used to analyse the information registered by the sensors, like the time instant in which an event occurs and, hence, this procedure will be used only to align the signal to maximize the correlation.

### 3.5. Mahalanobis Distance

In complex phenomena such as the evolution of the bike riding, the identification of threshold values is not unique among different users. Therefore, we preferred to apply an outlier detection as criteria to identify critical events. The use of Mahalanobis’ distance returned good results for the identification of outliers in a previous study related to motorcycle traffic conflict analysis [44]. The Mahalanobis’ distance identifies observations that lie far away from the center of the data cloud, giving less weight to variables with large variances or to groups of highly correlated variables. Given a multivariate vector $x={\left(x_{1},x_{2},\ldots ,x_{n}\right)}^{T}$, a relative vector of mean values $\mu ={\left(\mu_{1},\mu_{2},\ldots ,\mu_{n}\right)}^{T}$ and the covariance matrix *S*, the Mahalanobis distance is defined as [45]:

(3) $$D_{M}\left(x\right)=\sqrt{{\left(x-\mu \right)}^{T}S^{-1}{\left(x-\mu \right)}^{T}}$$

## 4. Results

In this section, the results related to the previous signal processing steps are depicted in two different scenarios: the first one is a calibration test performed to tune the processing procedure, the second scenario is related to an application of the before discussed procedure in a real riding.

### 4.1. Calibration Test

**Figure 3:**
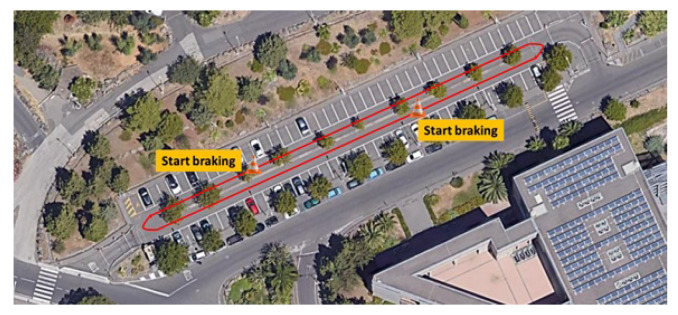
Satellite view of the calibration route.

A calibration test, labelled as C1, was carried out in a close route with an approximate length of 300 m in the University of Catania’s campus, as illustrated in Figure 3. It is located on flat ground and with good pavement condition. During the experiment, the biker was instructed ride at different speeds, applying braking manoeuvres with different deceleration rates. Starting of the braking zones were identified with the positioning of road cones. Raw data, collected by the two devices, have been processed to clean the signals from the noise given by the measurement system.

**Figure 4:**
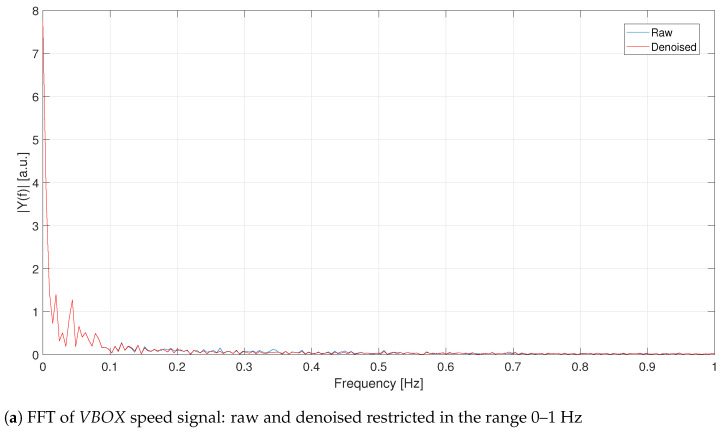

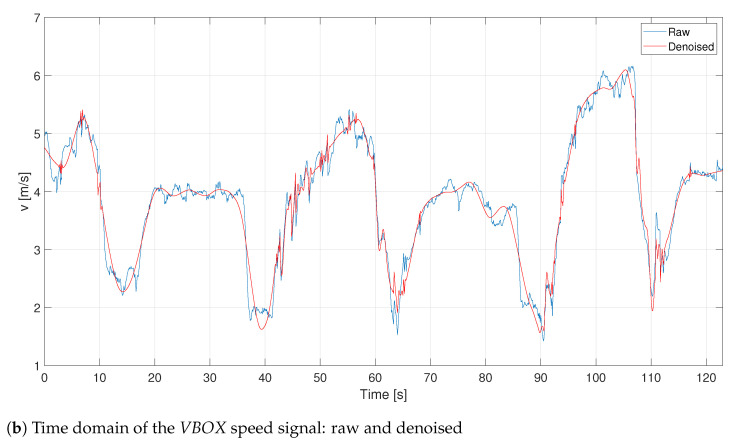
C1—Comparison between raw and denoised signals for the VBOX.

**Figure 5:**
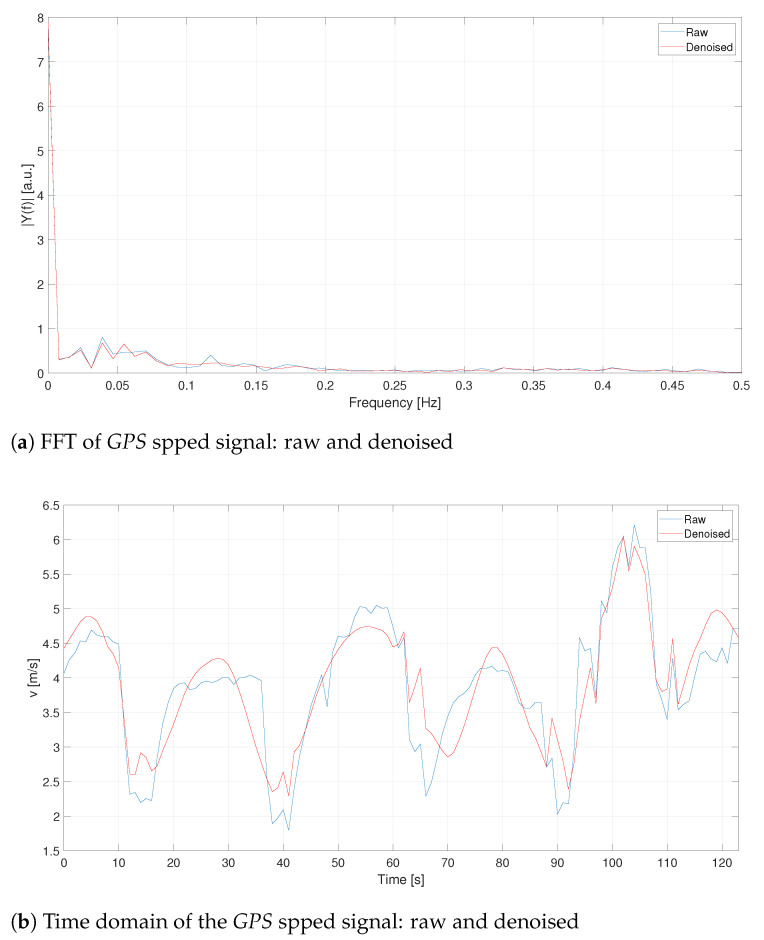
C1—Comparison between raw and denoised signals for the GPS.

**Figure 6:**
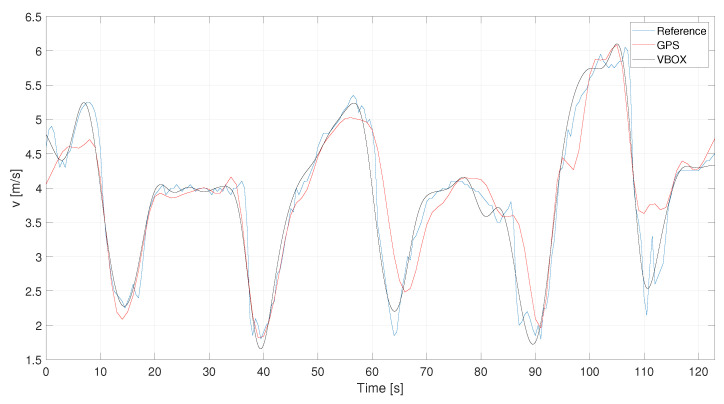
C1—Reference, GPS and VBOX speed signals.

Looking at the FFTs of Figure 4a and Figure 5a, it becomes evident that the more relevant information is contained at very low frequencies (less than 0.1 Hz). Both GPS1 and VBOX velocity signals are processed with a 5th order filter whose cut-off frequency has been set equivalent to 0.2 Hz, Figure 6. Such a filter allows to maintain only the informative contribution, neglecting the remaining noise left from the denoising procedure. Furthermore, the high order avoids signal attenuation because its amplitude is fundamental to detect traffic conflicts. As it is possible to see, remarkable improvements can be detected for the VBOX signal (both in time and frequency domains), while for the GPS the denoising procedure makes its trend worse, e.g., the constant trend from 20 s to 40 s is not maintained but approximated with a sinusoidal-like shape. This effect is probably due to its lower frequency with respect to VBOX one, as Figure 5a reveals.

**Figure 7:**
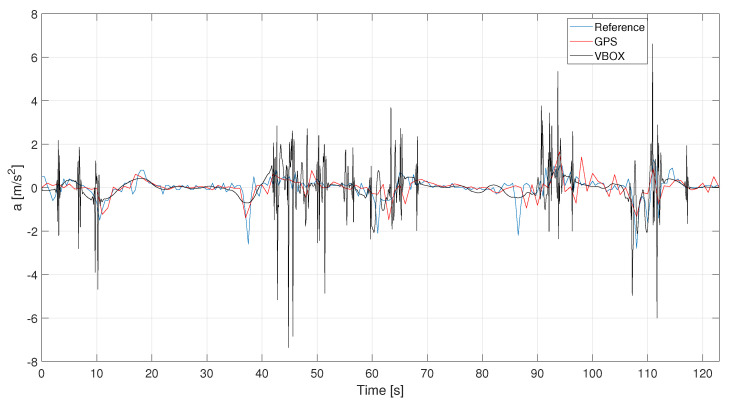
C1—Reference, GPS and VBOX acceleration signals.

The steps discussed above, i.e., denoising, outlier removal and filtering, are now applied for the acceleration signals. GPS and VBOX accelerations, derived from the speed signals, are evaluated and compared to the reference acceleration profile, as Figure 7 depicts. The accelerations are calculated as derivative of the raw velocities to avoid the useful information is lost during their calculations. Furthermore, for sake of visualization, the VBOX raw acceleration signal has been denoised according to previous discussed.

**Figure 8:**
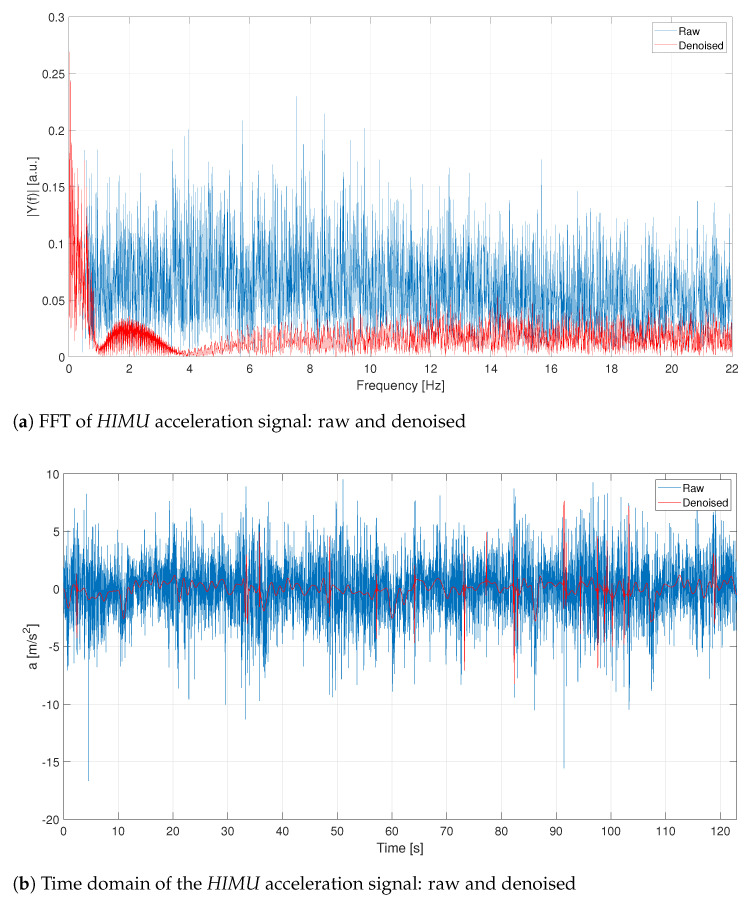
C1—Comparison between raw and denoised signals.

Besides, the HIMU acceleration signal, represented in Figure 8, is pre-processed according the steps introduced before. It is quite evident that the acceleration signal contains a lot of noise, also due to its high sampling frequency. Denoising and outlier removal have been applied to the HIMU signal and the related result is depicted in red. As shown in Figure 8, the signal has been strongly denoised. As for the GPS and VBOX signals, also the HIMU reveals that the most informative contribution is contained at very low frequencies.

**Figure 9:**
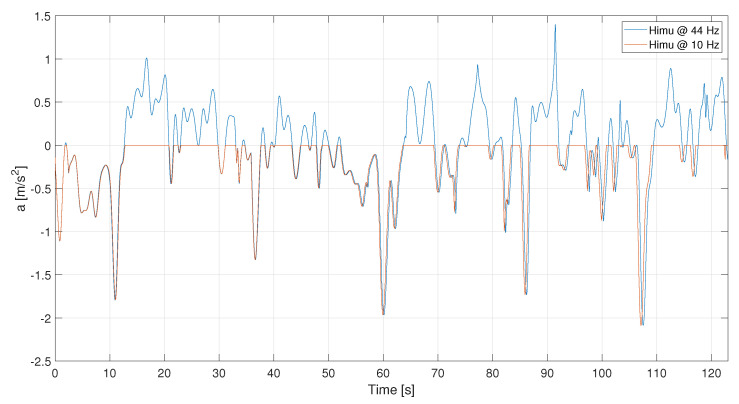
C1—HIMU acceleration signal resampled.

To correctly compare real acceleration signal (sampled at 2 Hz, so with a length of 2N×1) and the acquired sensors signals (GPS sampled at 1 Hz with a length of N×1, VBOX at 10 Hz with a length of 10N×1 and HIMU at 44 Hz with a length of 44N×1), a resampling procedure is required. It must be noticed that the downsampling, i.e., decimation, or upsampling, i.e., interpolation, do not add any further information but they allow to change the signals’ sampling frequency. A common sampling rate of 10 Hz has been fixed in order to keep the same length for all the four signals. The HIMU signal has been downsampled, while the GPS and the reference signals are upsampled. In Figure 9, an example of the resampling procedure is applied for the HIMU acceleration signal (filtered at $\omega_{n}$ = 0.5 Hz and n=1), looking at only its negative trend. Some misalignments in the resampled signal are due to a non-constant sampling rate in the signal acquisition. However, this problem is fixed in the following.

**Figure 10:**
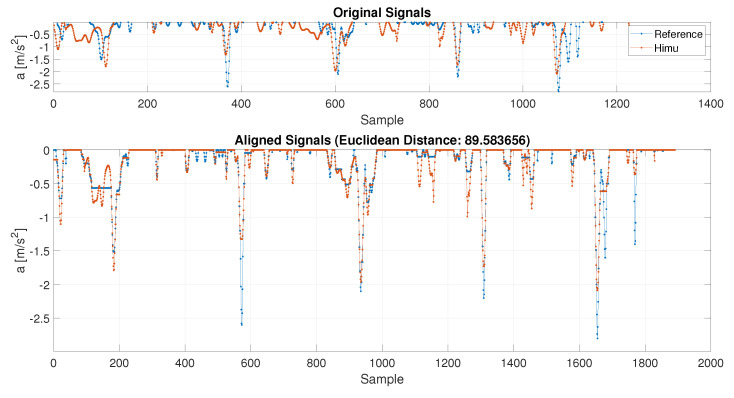
C1—DTW between reference and HIMU acceleration signals.

Once that all signals have been resampled at the same length, a peak alignment procedure has been performed with DTW procedure, Figure 10.

**Figure 11:**
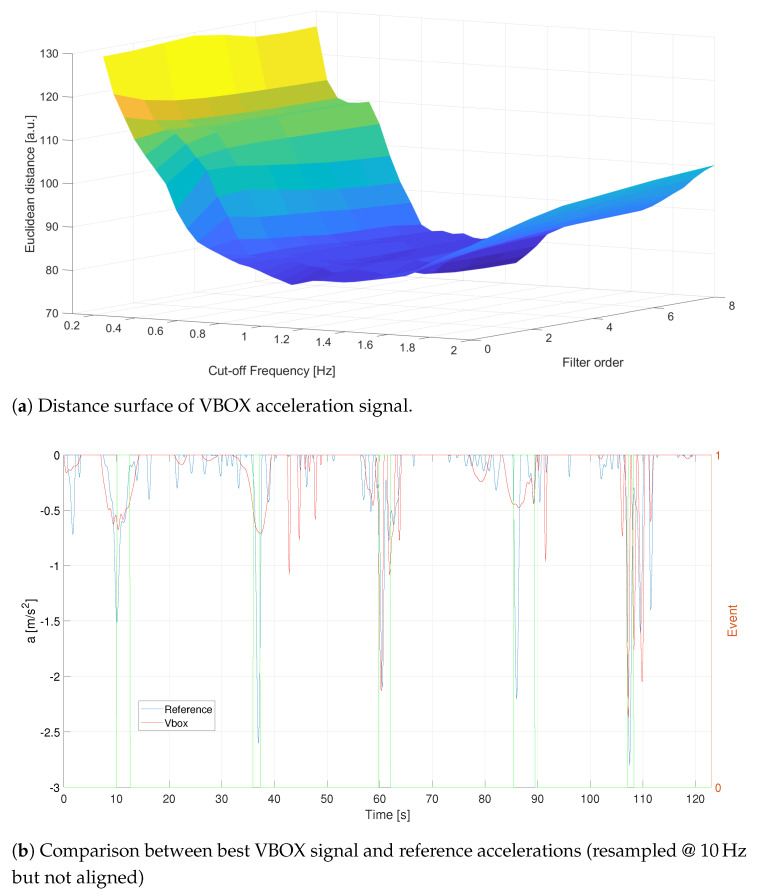
C1—Distance analysis results for VBOX. Events are reported as pulsed train (green solid line).

Once the sensor acceleration signals are opportunely preprocessed (denoised, outlier removed, filtered and resampled), it is now possible to find the best configuration of the filtering parameters $\left(\omega_{c};n\right)$ that minimize the Euclidean distance, evaluated with the DTW procedure, among the reference acceleration, $a_{ref}$, and those ones detected by VBOX, GPS1 and HIMU sensors, respectively $a_{VBOX}$, $a_{GPS1}$ and $a_{HIMU}$. In particular, the interval of the cut off frequency $\omega_{c}$ has been set between 0.1Hz–2Hz with an increasing step equal to 0.05, whereas the filter order changes between 1 and 8 with an increasing step equal to 1. In the case of VBOX, Figure 11, the best configuration is achieved with an order equal to n=7 and a cut-off frequency equal to $\omega_{c}$ = 1.05 Hz. These parameters allow to reach a minimum distance equal to $d_{a_{ref};a_{VBOX}}=74.8749$. As it is possible to see in Figure 11b, the VBOX acceleration follows almost faithfully the reference signal: obviously the VBOX signal considers all the minimum decelerations inside the reference profile.

**Figure 12:**
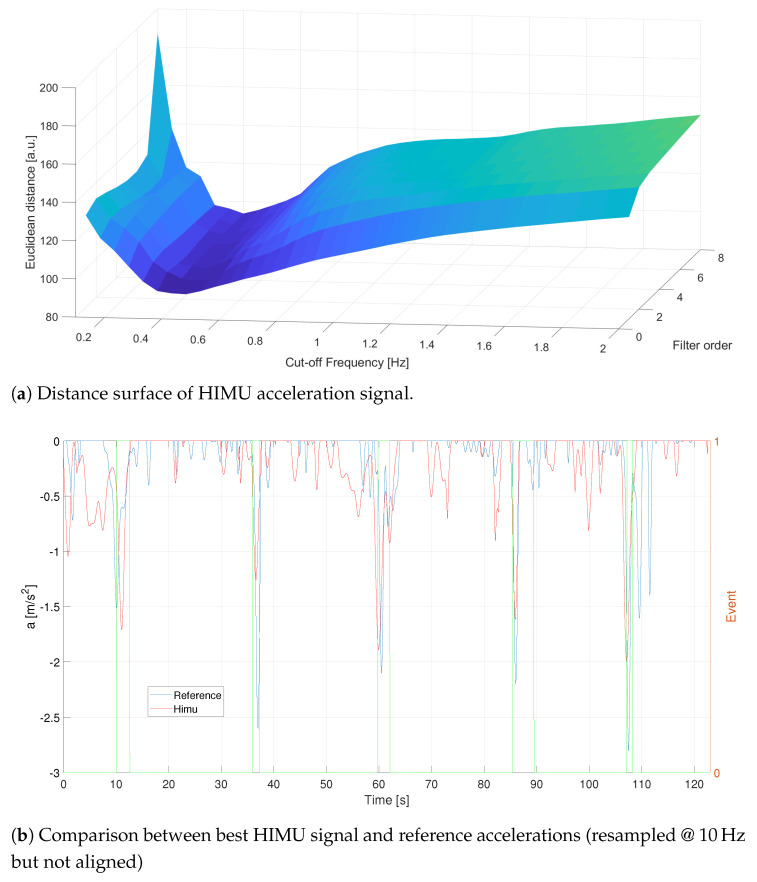
C1—Distance analysis results for HIMU. Events are reported as pulsed train (green solid line).

The results obtained for the HIMU signal are depicted in Figure 12. In this case the maximum correlation coefficient, $d_{a_{ref};a_{HIMU}}=87.8961$, is achieved with a filter order equal to n=1 and a cut-off frequency equal to $\omega_{c}$ = 0.45 Hz. In Figure 12b the filtered signal is reported, and it is possible to notice that HIMU follows, almost faithfully, the braking occurred during the acquisition.

**Figure 13:**
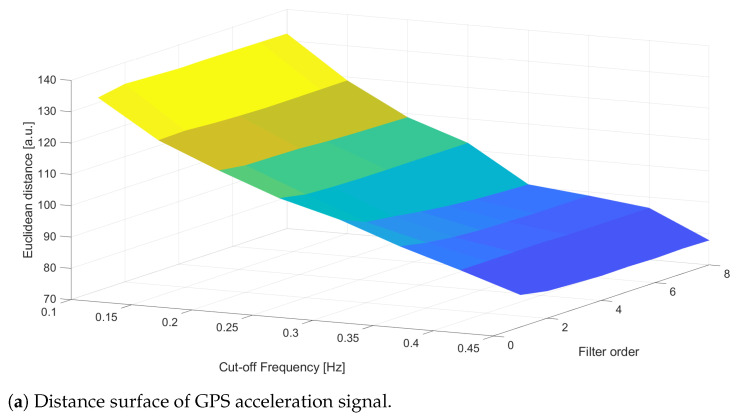

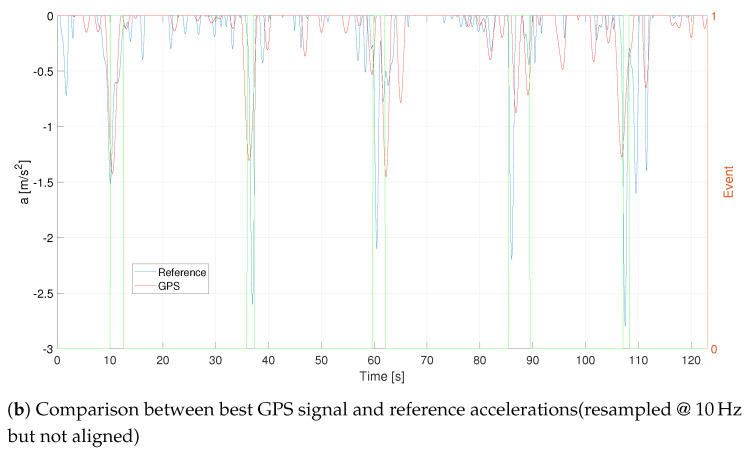
C1—Distance analysis results for GPS. Events are reported as pulsed train (green solid line).

Finally, the results for the GPS signal are represented in Figure 13, where the minimum Euclidean distance, $d_{a_{ref};a_{GPS1}}=77.7906$ is achieved with a filter order equal to n=6 and a cut-off frequency $\omega_{c}$ = 0.45 Hz. It must be noticed that, to minimize the Euclidean distance that follows the reference peaks, the signal is not filtered (the maximum cut-off is 0.5 Hz), as Figure 13b confirms.

**Figure 14:**
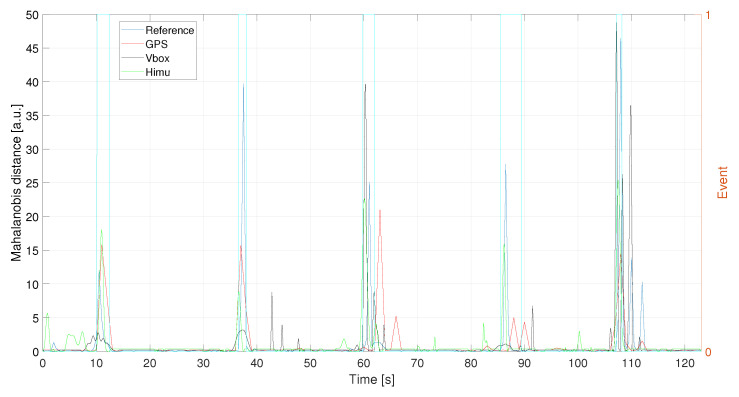
C1—Mahalanobis distances for Reference, GPS, VBOX and HIMU acceleration signals. Events are reported as pulsed train (light blue solid line).

In conclusion, the Mahalanobis distances, computed for each best signal, are depicted in Figure 14: VBOX and HIMU give best results in terms of both event identification and its severity, while the GPS, considering also its lower sampling frequency, is able to detect all the traffic conflicts reported in this case study.

### 4.2. Real Scenario

**Figure 15:**
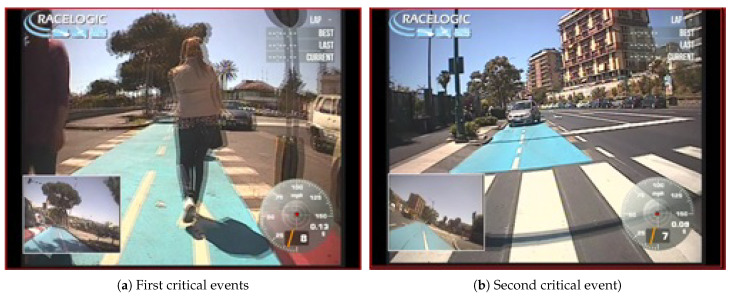
L1—Critical events occurred during the riding.

Data were collected with the instrumented bike in a cycle track, a two-way bike lane, 2.0 m wide and 2.4 km long, separated by a curb from the normal lane. The event classification was carried out by direct observation of the video that was recorded during the test using the “Video VBOXLite” equipment and tool and the correspondent speed and acceleration profiles. A TC was defined as the interaction between the cyclist and another user moving in the opposite direction of the bicyclist requiring the bicyclist to perform an evasive manoeuvre (i.e., braking, swerving) irrelevant of the severity level of the manoeuvre. Two different safety critical events were detected along the route during the ride, see Figure 15: in the first one, Figure 15a the traffic conflict was with a pedestrian with event temporal of 0.8 s while the second, Figure 15b, was with a car with event temporal of 0.9 s.

**Figure 16:**
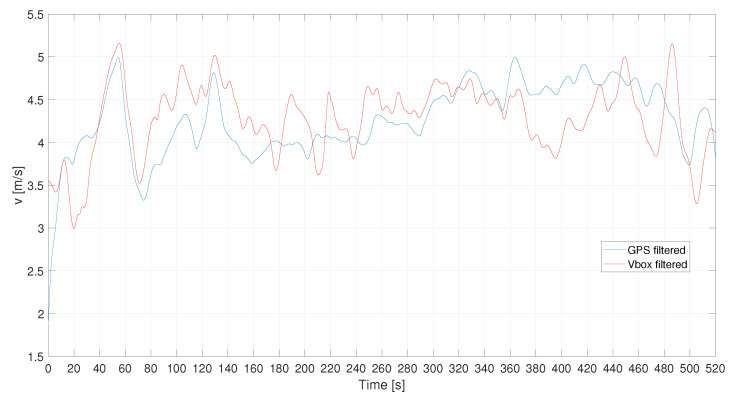
L1—VBOX and GPS velocity filtered signals.

In Figure 16 the filtered speed of this case study is reported. Also, in this case, the denoising procedure has not been applied to the GPS signal because it changes the signal trend. These signals have been filtered using a cut-off frequency equal to $\omega_{c}$ = 0.05 s and n=1.

**Figure 17:**
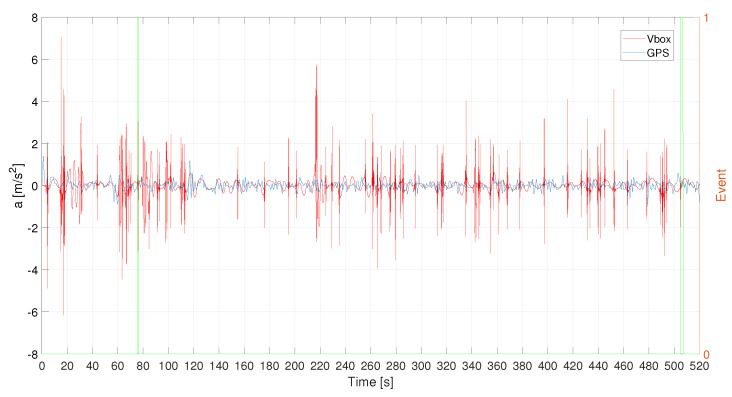
L1—VBOX and GPS acceleration raw signals. Events are reported as pulsed train (green solid line).

**Figure 18:**
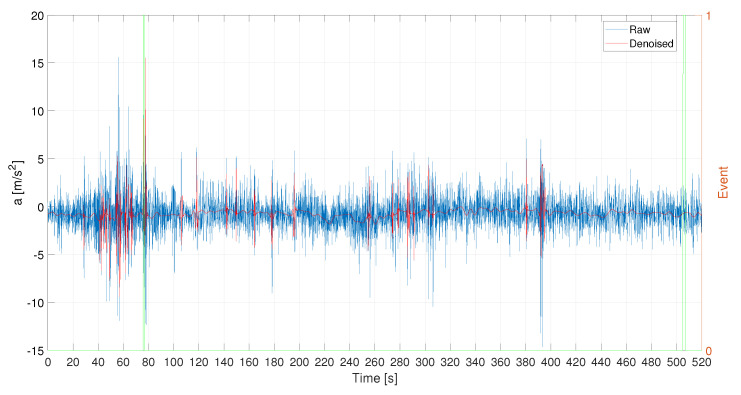
L1—HIMU acceleration signals: raw and denoised. Events are reported as pulsed train (green solid line).

In Figure 17 and in Figure 18 the raw acceleration signals for the three different acquisition systems. Also, in this case, the denoising procedure is necessary for the HIMU signal because it is very noisy.

**Figure 19:**
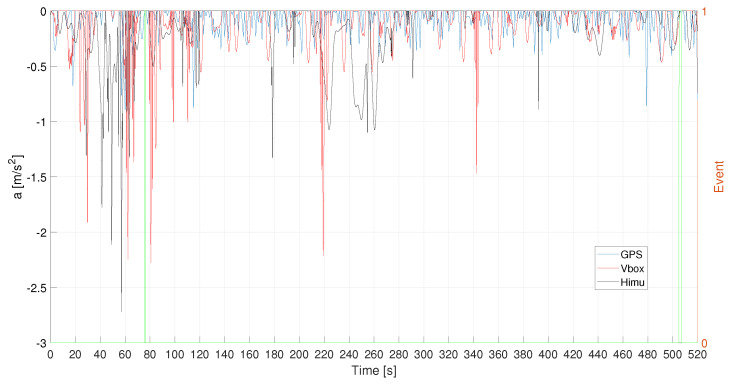
L1—Filtered acceleration signals. Events are reported as pulsed train (green solid line).

**Figure 20:**
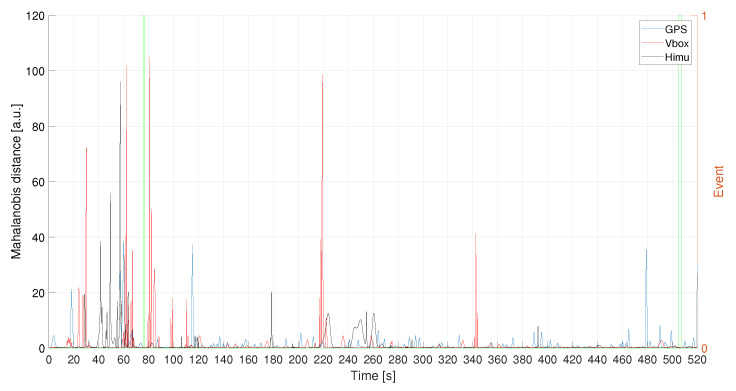
L1—Mahalanobis distance for the acceleration signals. Events are reported as pulsed train (green solid line).

Evaluating the accelerations profiles for each of the three recording systems, they have been filtered according to the obtained values of the procedure discussed in the previous section, Figure 19. In particular, HIMU signal has been opportunely denoised in order to deal with a better signal before the filtering phase. The Mahalanobis’ distance has been evaluated for the filtered signal, as depicted in Figure 20. It is possible to notice that each of the three system is able to detect traffic conflicts.

## 5. Conclusions and Discussion

In this study, a signal processing procedure has been proposed in order to correctly deal with speed and acceleration signals to recognize hard breaking. The aforementioned procedure allows to manage high-noisy signals, such as the smartphone accelerations with standard filters that are not opportunely cleaned. Additionally, the Dynamic Time Warping has been exploited in order to correctly compare signals with different time rate. Furthermore, the proposed methodology has been applied for validation in real scenario with actual traffic conflicts between cyclist and other road users. Analyzing the obtained results, it can be noticed that a higher sampling frequency allows to detect and evaluate the severity of detected traffic conflicts, which raises due to a higher number of samples. As drawback, exploiting a high sampling frequency leads to a more storage and battery requirements, which are constraints in Big-Data applications. However, results have also proved that using smaller sampling frequency can detect traffic conflicts, requiring a simpler hardware setup and less memory storage. This approach, thanks to its minimal hardware setup, allows all user to collect acceleration data, hence, detect and notify critical zones for cyclists. Anyway, dataset available from GPS probe data providers (e.g., STRAVA, bike sharing) are typically collected with a 5–25% capture rate (i.e., less than 0.25 s) making such data not suitable for traffic conflict studies rather than more general roadway analytics like O/D and route mapping. The outlier detection technique based on Mahalanobis’ distance outperform traditional approach based on threshold values to identify the critical events in terms of both event identification and severity.

Further research activities will be focused on the increasing of the dataset in order to define new techniques to classify the traffic conflict severity for low-frequency sampled signals.

## Data Availability

The data presented in this study are available on request from the corresponding author. The data are not publicly available due to a Non-Disclosure Agreement.
